# Simethicone for the Preparation before Esophagogastroduodenoscopy

**DOI:** 10.1155/2011/484532

**Published:** 2011-08-04

**Authors:** Majid Ahsan, Leila Babaei, Ali Gholamrezaei, Mohammad Hassan Emami

**Affiliations:** ^1^Medical Student Research Center, Isfahan University of Medical Sciences, Isfahan, Iran; ^2^Poursina Hakim Research Institute, P.O. Box 81465-1798, Isfahan 8158844771, Iran; ^3^Department of Gastroenterology, Isfahan University of Medical Sciences, Isfahan, Iran

## Abstract

*Aim*. The presence of air bubbles and foam in stomach and duodenum is a common problem during esophagogastroduodenoscopy (EGD). *Methods*. Candidates of elective EGD received 40 mg chewable tablet of simethicone (*n* = 90) or placebo (*n* = 83), with 30 mL water, 15–30 min before the EGD. Foam/air bubbles during endoscopy were assessed and graded on a 4-point scale, and patients' satisfaction with the endoscopy was scored from 0 to 10. *Results*. The amount of gastric but not duodenal foam/air bubbles was significantly lower in the simethicone group compared with the placebo group (*P* = 0.002). Duration of endoscopy was, on average, one minute shorter in the simethicone group compared with the placebo group (*P* < 0.001). Patients' satisfaction with the procedure was the same in the two groups. *Conclusion*. Administration of simethicone prior to EGD reduces the amount of gastric foam and bubbles and provides better visibility for evaluating the mucosa. It also decreases the duration of endoscopy. Further trials are required to find the final effect of the drug on diagnosis of pathological lesions.

## 1. Introduction

Upper gastrointestinal endoscopy (esophagogastroduodenoscopy or EGD) is one of the most common diagnostic and therapeutic methods of upper gastrointestinal diseases [1]. One limitation of the method is, however, the presence of air bubbles and foam in stomach and duodenum, such that it is difficult or sometimes impossible for a gastroenterologist to evaluate the mucosa using the images obtained in the presence of the bubbles. This will lead to decreased diagnostic accuracy, prolonged endoscopy time, and decreased patient's tolerance [2]. Therefore, gastric and intestinal preparation prior to endoscopy is necessary for the removal of the bubbles.

An appropriate preparation method should be able to remove the bubbles, not having side effects, be tolerable for patients, and be applicable for most patients in different conditions [3]. Currently, except fasting prior to endoscopy, no standard method has been recommended for prior EGD preparation. Although some specialists routinely administer simethicone for endoscopy preparation [4], according to the searches performed in medical databases, until conducting the present study, there was no systematic study on simethicone with respect to its effects and side effects, and also the potential of the drug to be recommended for all patients. 

Simethicone is a detergent, which is a chemical mixture of dimethyl polysiloxane and silica gel. It is physiologically inactive and nontoxic. It can be taken orally and cannot be absorbed through gastrointestinal system [5]. By reducing the adhesion force of air bubbles, simethicone removes the bubbles. Thus, it is expected that the drug can be used for removing gastric and duodenal foams and bubbles [5]. Simethicone does not have any known drug interaction, and no significant complication has been reported for it. Therefore, the drug has been used for treatment of patients with vague abdominal complaints (because of large amount of gases), and positive effects have been observed [6]. Moreover, the drug (in solution formulation) has been used in some studies for intestinal preparation prior to colonoscopy and capsule endoscopy [7]. However, thus far, no reliable report for routine use of the drug in preparation prior to EGD was available. Thus, we have evaluated the effect of simethicone on the reduction of foam and air bubbles during upper gastrointestinal endoscopy.

## 2. Methods

This randomized, placebo-controlled, double-blinded (patients and physician), clinical trial was carried out on patients above 18, who referred to Poursina Hakim Clinic, Isfahan (IRAN), for elective EGD. Considering the study power as to be 80% and determining the type I error as 0.05, and also setting the minimum expected difference in average of foam/air bubbles between the two groups as 0.4, according to the available data [8], and also assuming 10% for missing probability, the sample size for each group determined to be 80. The study was approved by the Ethical Committee of Isfahan University of Medical Sciences, and all patients signed an informed written consent.

The sampling was carried out subsequently, and the participants were assigned to the simethicone or placebo groups randomly (according to the computer-generated table of random numbers) [9]. Fifteen to 30 minutes before EGD, in the presence of the researcher, the patients chewed a simethicone (40 mg) or placebo tablet, and then took 30 mL of water. Shape, box, and the route of administration of the drug were the same for the two groups. The placebo was prepared in the Pharmacy School of Isfahan University of Medical Sciences from corn starch and in the form and color of simethicone tablet.

Regarding the previous studies [10, 11], we used a 4-point scale to separately measure the amount of gastric and duodenal foam/air bubbles; (0) no air bubbles, (1) there was a small amount of bubbles, without interfering in the evaluation, (2) there was a considerable amount of air bubbles and foam, such that it was somehow difficult to evaluate, and (3) mucosal evaluation was hardly possible owing to the presence of foam and air bubbles. The amount of foam and air bubbles was recorded immediately at the end of endoscopy procedure by the gastroenterologist. The duration of the endoscopy procedure was also measured and recorded. If there was a stenosis in the upper gastrointestinal tract, such that gastric or duodenal evaluation was not possible or the stenosis led to a prolonged procedure, the patient was excluded from the study. Moreover, after carrying out the procedure, the participants' satisfaction with the endoscopy procedure was scored on a numerical scale, in which 0 showed the least satisfaction level and 10 showed complete satisfaction. The data was analyzed by SPSS software, v. 16.0, using Chi-square test and independent sample *t*-test. In case of lack of normal distribution for quantitative data, we used Mann-Whitney test for comparison of the two groups.

## 3. Results

**Figure 1:**
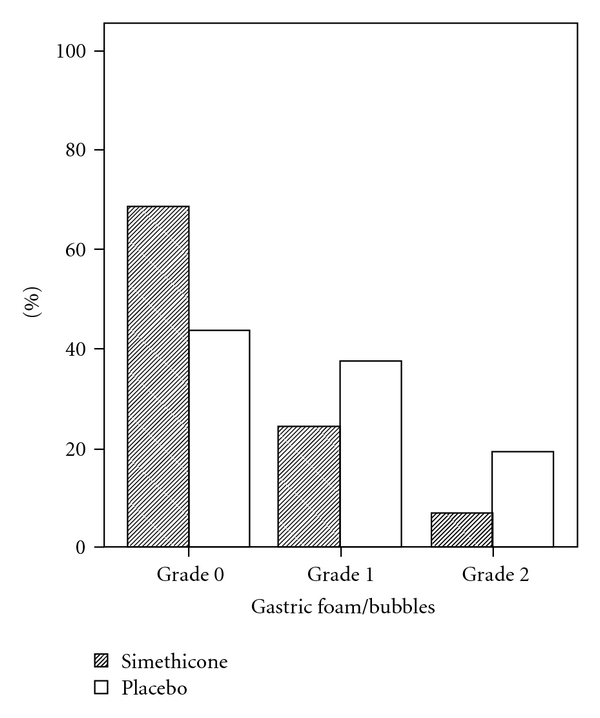
Comparison of the amount of gastric foam/air bubbles between the two groups.

**Figure 2:**
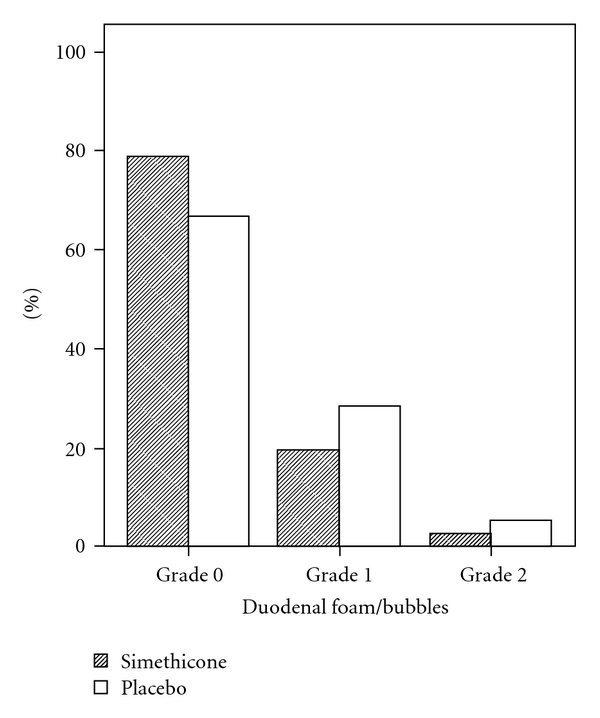
Comparison of the amount of duodenal foam/air bubbles between the two groups.

We included 90 and 83 patients in the simethicone and placebo groups, respectively. The two groups were similar in terms of age and sex. Comparison of the amount of gastric and duodenal foam/air bubbles in the two groups is demonstrated in Figures 1 and 2. In the two groups, the amount of gastric and duodenal foam/air bubbles was limited to grade 2. The amount of gastric foam/air bubbles was significantly lower in the simethicone group, compared with that of the placebo group (*P* = 0.002). However, the two groups were not significantly different with regard to the amount of duodenal foam/air bubbles (*P* = 0.422). 

**Figure 3:**
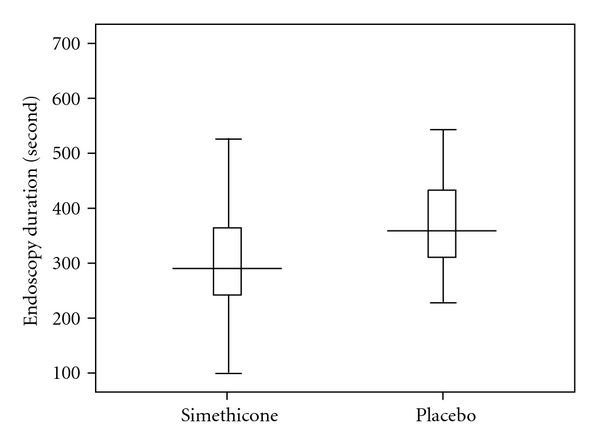
Comparison of the endoscopy duration between the two groups.

Comparison of the two groups with respect to the duration of endoscopy procedure is demonstrated in Figure 3. The duration of endoscopy procedure was one minute shorter in the simethicone group (308.0 ± 116.2 versus 376 ± 108.1 seconds, *P* < 0.001). 

**Figure 4:**
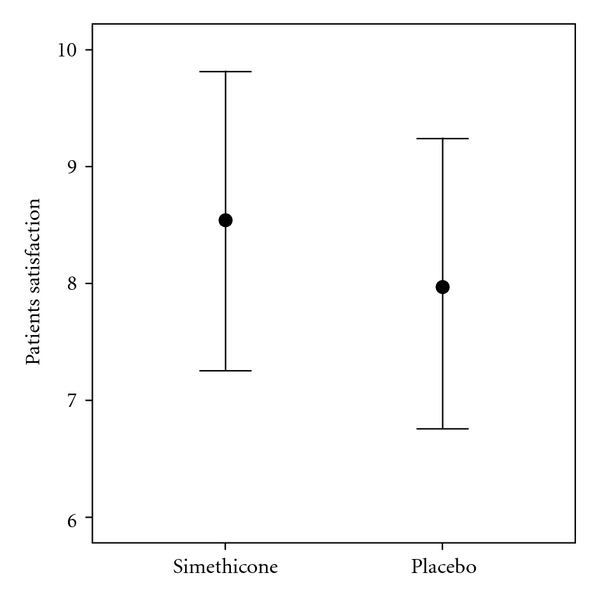
Comparison of the patients' satisfaction with the procedure between the two groups.

Comparison of the two groups with respect to the patients' satisfaction with the endoscopy procedure is shown in Figure 4. The two groups were not significantly different in this regard (*P* = 0.646). 

We did not observe any severe medical complication like allergic reactions in the simethicone group. 

## 4. Discussion

The aim of the study was to determine the effectiveness of simethicone in the preparation prior to EGD. According to the results obtained, simethicone decreased the amount of gastric air bubbles and foam significantly but has no significant effect on the amount of duodenal air bubbles and foam. Another noteworthy finding was the decrease in the endoscopy procedure duration by one minute on average in the simethicone group. However, simethicone did not affect the patients' satisfaction with the procedure. Lack of significant effect on duodenal air bubbles and foams can be due to the small amount of duodenal air bubbles during the procedure, such that only 2.4% of all patients had grade 2 of air bubbles, and, therefore, a larger sample size is required to evaluate the effect of simethicone in this respect. In addition, if we want the drug reaching duodenum, a larger amount of water is required to be taken with the drug. Moreover, in timing the drug administration, we should take into account the lagging in emptying gastric content into the duodenum. 

According to our literature review, there was only one report available on the effectiveness of simethicone versus placebo in preparation prior to EGD, and most studies evaluated the effectiveness of adding simethicone to the colonoscopy or capsule endoscopy preparation regimens [7]. In the study carried out by Keeratichananont et al., 121 candidates of EGD received simethicone (2 mL) or placebo solution with 60 mL of water, 15 to 30 minutes prior to EGD. They observed a decrease in the amount of esophageal, gastric, and duodenal air bubbles and foam in the simethicone group. Moreover, patients and physicians in the simethicone group were more satisfied with the procedure. However, the drug did not influence the duration of endoscopy procedure [12]. Therefore, it seems that if we want the sufficient amount of drug to reach the duodenum, a larger amount of water should be taken with the drug. According to the review and meta-analysis carried out by Wu et al. on 13 placebo-controlled studies on the effectiveness of simethicone in capsule endoscopy and colonoscopy, simethicone significantly decreases the amount of air bubbles and foams in the endoscopy field leading to an increase visibility. This provides a better chance for more accurate evaluation of the mucosa. However, the final effect of the drug on the diagnosis of pathological lesions is not investigated yet [7].

In spite of being placebo-controlled, and also randomization, and an appropriate sample size, the present study had some limitations. The amount of air bubbles and foam was evaluated by a single person (the endoscopist), on a 0–4 scale. It was better to carry out the evaluation on the basis of image processing and quantitative scales, to completely remove measurement errors and biases. Furthermore, it is not known to what extent a decrease in the amount of air bubbles and foam, and an increase in the quality of mucosal images will improve diagnosis of pathological lesions during endoscopy. Further studies are required in this respect.

## 5. Conclusion

Using simethicone prior to upper gastrointestinal endoscopy significantly reduces the amount of air bubbles and foam and increase the visibility during the procedure. This provides the possibility of more accurate evaluation of the mucosa and also decreases the endoscopy duration. We suggest comparison of effectiveness of different doses, timing of administration, and volume of simultaneous liquid, as well as the final effect of the drug on diagnosis of pathological lesions in future studies.
